# Efficient inference, training, and fine-tuning of protein language models

**DOI:** 10.1016/j.isci.2025.113495

**Published:** 2025-09-03

**Authors:** Muhammed Hasan Çelik, Xiaohui Xie

**Affiliations:** 1 Department of Computer Science, University of California, Irvine, Irvine, CA, USA; 2 Center for Complex Biological Systems, University of California, Irvine, Irvine, CA, USA

**Keywords:** Protein, Bioinformatics

## Abstract

Protein language models (PLMs) have shown great promise in protein structure and function predictions, but their adoption is limited by computational cost. We address this challenge by enhancing the efficiency of evolutionary scale modeling (ESM). Using FlashAttention and sequence packing, we achieve 4–9× faster inference and 3–14× lower memory usage. Four-bit quantization of billion-parameter models further reduces memory by 2–3× while preserving accuracy for missense variant effect prediction. Training is also optimized, cutting runtime 6-fold with methods, such as activation checkpointing and DeepSpeed zero-offload. Parameter-efficient fine-tuning of a few adapter weights yields state-of-the-art performance at protein property and function predictions, resulting in 70% Spearman’s correlation for melting point and 87% AU-PRC for transcription factor identification. Our efficient ESM (ESME) implementation significantly lowers the barrier to using these powerful models, making them accessible to academic laboratories with limited computational resources. The code is available on GitHub.

## Highlights

• FlashAttention and sequence packing enable 4–9× faster inference and 3–14× lower memory usage
• Introducing weight quantization reduces memory usage by 2–3× with minimal accuracy loss
• Training runtime is reduced 6-fold using activation checkpointing and zero-offload
• Parameter-efficient fine-tuning achieves state-of-the-art performance in function prediction

## Graphical abstract

**Figure:**
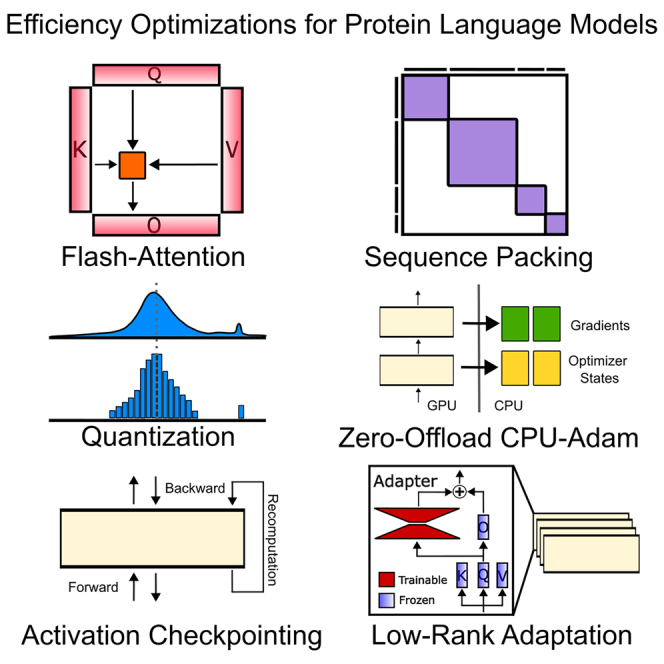

## Introduction

An in-depth understanding of protein properties and function is crucial for the discovery of the mechanisms underlying essential cellular processes.^1^ Deep learning techniques have long been employed for various in-silico protein prediction tasks^2^ and recently led to breakthroughs in structure prediction.^3^^,^^4^^,^^5^^,^^6^^,^^7^ Furthermore, protein language models (PLMs) enable the prediction of protein properties,^8^^,^^9^ function,^10^ and the effect of the missense variants on protein fitness and disease.^11^^,^^12^^,^^13^ Recent advances have been achieved by the adaptation of large language models (LLMs) based on the transformer architecture from natural language processing (NLP)^14^^,^^15^ to the proteomics field.^9^^,^^16^^,^^17^^,^^18^^,^^19^^,^^20^ These models, known as PLMs, leverage abundant protein sequence data, and are trained using semi-supervised objectives to predict masked amino acids from the surrounding protein sequence context.^21^^,^^22^ As a result, the attention mechanism in the transformer layers captures the complex interactions between the amino acids during the semi-supervised training to predict masked amino acids. Traditional sequence-based alignment methods can capture pairwise conservation between amino acids; however, modeling higher-order interactions is computationally intractable and requires heuristic approximations.^23^ In contrast, deep learning models capture high-order epistatic interactions and context-dependent sequence constraints.^24^^,^^25^ Thus, PLMs can perform a wide range of downstream tasks, including protein structure prediction.^3^^,^^6^

PLMs facilitate protein prediction tasks, particularly when supervised training data are limited. These models are fine-tuned to predict biochemical properties by leveraging predicted structures and contextualized sequence embeddings. For example, after fine-tuning with a small number of additional training samples, PLMs can accurately predict various biochemical properties, such as folding stability^22^^,^^26^^,^^27^ and melting point.^28^ By capturing intrinsic features and conserved amino acids, PLMs enable effective generalization through few-shot learning. Beyond structural predictions, PLMs also infer biophysical features, such as protein dynamics in disordered regions^29^ and phase transitions.^30^ Since the properties of proteins determine their functions, PLMs are also highly effective in predicting the functional characteristics of proteins. For instance, PLMs can accurately predict gene ontology (GO) terms^10^ and enzyme commission (EC) numbers^31^^,^^32^ directly from amino acid sequences. Furthermore, the prediction of protein-protein and protein-small molecule interactions^5^ is crucial for understanding the role of proteins in biological processes, and PLMs can predict these interactions, including ligand-binding proteins,^33^ peptide-T cell receptor interactions,^34^ and drug-protein interactions.^35^^,^^36^^,^^37^ Finally, PLMs have important applications in protein design, enabling the generation of amino acid sequences with specified characteristics,^38^^,^^39^ thereby facilitating computational approaches in drug development.

Another application of PLMs is the variant effect prediction of missense variants.^11^^,^^12^^,^^13^ Classifying deleterious missense variants from benign ones is critical for the identification of pathogenic variants in disease studies. The PLMs learn the natural sequence space of the proteins selected by evolution; thus, they can estimate the variants disrupting the protein fitness. Learning the sequence distribution of homolog proteins and the co-conservation of amino acids reveals the epistatic interactions and improves variant effect prediction.^24^^,^^25^^,^^40^

A significant challenge in utilizing PLMs is their substantial computational cost, with training requiring thousands of GPU hours on large computing clusters.^41^ This high computational cost makes training PLMs from scratch impractical, especially for researchers relying on academic laboratory-grade computation infrastructure, thereby limiting the ability to experiment with different modeling choices and hindering progress in research. Furthermore, inference and fine-tuning of these models are computationally expensive; our initial experiments revealed that inference on long protein sequences often results in memory errors. Fine-tuning, which requires backpropagation, further increases memory requirements. The large memory footprint also constrains the batch size during fine-tuning, which is essential for stable training and objectives like contrastive learning. Recent advances in efficient inference,^42^ fine-tuning,^43^ and training methods^44^^,^^45^ in the LLMs literature offer a promising solution to these limitations; thus, adapting these techniques could also facilitate the broader use of PLMs.

In this work, we implemented techniques to enhance the efficiency of PLMs and benchmarked their performance across various tasks, including variant effect prediction, using our reimplementation of the widely used evolutionary scale modeling (ESM) family, including ESM1v, ESM1b, ESM2, and ESM-Cambrian (ESM-C) models. We integrated widely used techniques like Flash-Attention^46^ and sequence packing to efficiently handle proteins of variable lengths, leading to a substantial reduction in both inference and training time, as well as memory usage. These reimplemented models achieve performance comparable to the original models in missense variant effect prediction based on the ProteinGym benchmark dataset,^47^ confirming the correctness of our implementation. Moreover, we achieve state-of-the-art performance on protein properties on the fitness landscape inference for proteins (FLIP) benchmark dataset^28^ and function prediction by only training a small fraction of model weights. Overall, the approaches employed in this work greatly improve the efficiency of PLMs and enable their use on academic computing infrastructure. Our open-source, efficient implementation ESME is available on PyPI (pypi.org/project/esm-efficient), GitHub (github.com/uci-cbcl/esm-efficient), and HuggingFace (huggingface.co/mhcelik/esm-efficient).

## Results

We applied a combination of efficient LLM techniques to the ESM models, chosen for their standard transformer architecture, which facilitates the easy adaptation of LLM methods from NLP. We conducted in-depth benchmarking of the original ESM and efficient ESME reimplementation for inference, training, and downstream fine-tuning tasks (Figure S1).

### Benchmarking the model inference

**Figure 1:**
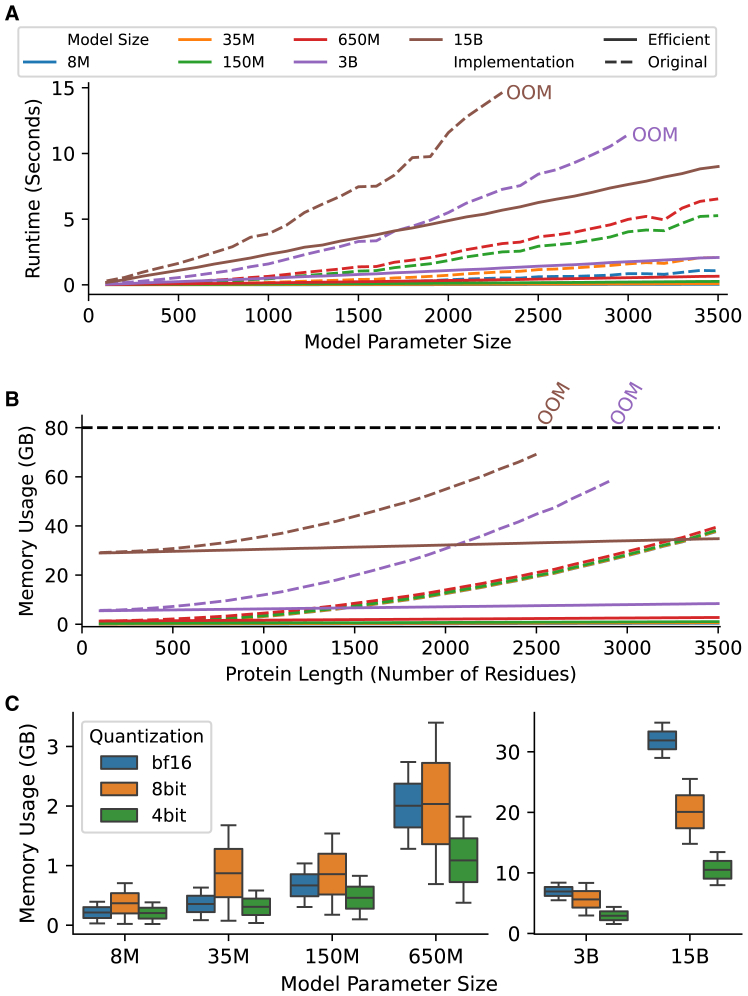
The benchmark of inference performance (A) The runtime comparison between the original ESM2 model (solid line) and ESME2 (dashed line) demonstrates the reduction in runtime as protein length increases (batch size of 16 proteins in subfigures A–C). M and B are abbreviations for million and billion, respectively. (B) The benchmark of memory usage shows that FlashAttention scales linearly with sequence length, while dot-product attention scales quadratically. (C) The model quantization techniques effectively reduce memory usage, but their impact is significant only in billion-scale models.

The first technique we leveraged in the efficient ESME implementation is FlashAttention.^46^ FlashAttention is an efficient, IO-aware self-attention algorithm that optimizes memory usage and runtime for long sequences by minimizing read/write operations between high-bandwidth memory (HBM) and SRAM while fusing operations to reduce memory overhead (STAR Methods). To demonstrate performance gains, we sampled proteins of varying lengths from UniProtKB/Swiss-Prot^48^ and measured runtime and memory usage. Inference performance was benchmarked on NVIDIA A100 GPUs using ESM2 models ranging from 8 million to 15 billion parameters, with a batch size of 16 proteins in brain floating point (bfloat16)^49^ for both original and efficient implementations. ESM1v and ESM1b were not included in this benchmark, as their behavior is comparable to the similarly sized ESM2 650M model. Adapting FlashAttention can reduce model runtime by ∼3–10 times across all model sizes, depending on protein length (Figure 1A; Table S1). For example, inference on a batch of 16 proteins with lengths of 300–400 residues takes ∼0.07 s with the ESME-2 650M model, compared to ∼0.21 s with the original implementation. The runtime of the models with FlashAttention scales linearly with input size, whereas the dot-product attention in the original implementation scales quadratically. Similarly, memory usage scales quadratically with input size in the dot-product attention implementation, limiting the batch size and inference on large proteins (Figure 1B; Table S2). Long protein sequences are often truncated to avoid an out-of-memory (OOM) error; however, FlashAttention maintains a small memory footprint even for large proteins. For instance, the 3-billion-parameter ESME-2 model uses approximately 5.6 gigabytes (GB) of GPU memory for a batch of proteins 300–400 amino acids long and no more than ∼8.4 GB for proteins up to 3,500 amino acids. In contrast, the original implementation cannot handle proteins longer than 3,000 amino acids for billion parameter models.

The proteins vary significantly in length; for example, ∼12% of human proteins are longer than 1000 amino acids, while the median protein is 414 amino acids long (Figures S2A and S2B). With linear scaling, the GPU memory usage of a batch of proteins can be quantified by the number of token (amino acid) count in the batch. Consequently, we engineered dataloaders that prepare batches based on token count rather than the number of proteins (Figure S3). To efficiently handle batches with varying protein lengths and maximize GPU utilization, we utilize sequence packing,^50^ which concatenates variable-length protein sequences into a single long chain without padding and utilizes attention masks to preserve batch structure (STAR Methods). Regardless of model size, FlashAttention with sequence packing can handle up to 100,000 tokens without memory errors (Figure S4). This combination ensures consistent computational load across batches, achieving full GPU utilization and enhancing model inference performance 9.4-fold for ESM2 and 3.8-fold for ESM-C models (Figure S5). The small footprint of FlashAttention and extending batch sizes using sequence packing could further enable training large PLMs on tasks involving protein complexes^51^ and even genome-scale protein predictions.^52^

We explored weight quantization techniques to optimize inference by benchmarking 8-bit quantization with LLM.int8()^53^ and 4-bit quantization using QLoRA^54^ algorithms using bitsandbytes package.^55^ Quantization provides marginal benefits for only billion-parameter models (3B and 15B), primarily by reducing memory usage through compressing model weights to lower precision (Figure 1C; Table S2). For instance, 4-bit and 8-bit quantization reduces memory usage to 41% and 80% of the unquantized inference for 3B models and 33% and 63% for 15B models. However, the memory overhead from the quantization constants needed for quantization and dequantization limits the effectiveness of these algorithms for sub-billion-parameter models. Additionally, 8-bit quantization introduces computational overhead, nearly doubling inference time, whereas 4-bit quantization achieves a runtime comparable to bfloat16 precision (Figure S6; Table S1).

### Efficient reimplementation replicates the pseudo-perplexity of original models

We use pseudo-perplexity^56^ as the primary metric to evaluate model uncertainty on given protein sequences. Pseudo-perplexity is computed by systematically masking each position in a sequence, one at a time, and estimating the probability of the masked token given the sequence context (STAR Methods). To validate the correctness of our efficient reimplementation, we compared the pseudo-perplexity of the original and replicated models across 500 randomly sampled human proteins. We use full precision (float32) for the model weights of the original models and bfloat16 for the efficient implementations, except for the ESMC models, where bfloat16 is used for both the original and efficient versions to utilize FlashAttention for both models. As expected, the ESM2-8M model with the smallest parameter size exhibited the highest pseudo-perplexity of 12.744 ± 3.033 (SD) for the original and 12.735 ± 3.027 for the efficient model. The ESM2-15B model with the largest parameter size achieved the lowest pseudo-perplexity of 3.637 ± 3.423 for the original and 3.636 ± 3.423 for the efficient model (Figure 1A; Table S3). The mean squared error (MSE) between original and efficient implementations was extremely small across all models (e.g., ESM1b: MSE = 0.0023, ESM2-150M: MSE = 0.0006, ESMC-300M: MSE = 0.0005), indicating that our reimplementation accurately reproduces the behavior of the original models. The replication of the pseudo-perplexities across proteins confirms the correctness of the efficient implementation.

### Benchmarking variant effect prediction on ProteinGym

**Figure 2:**
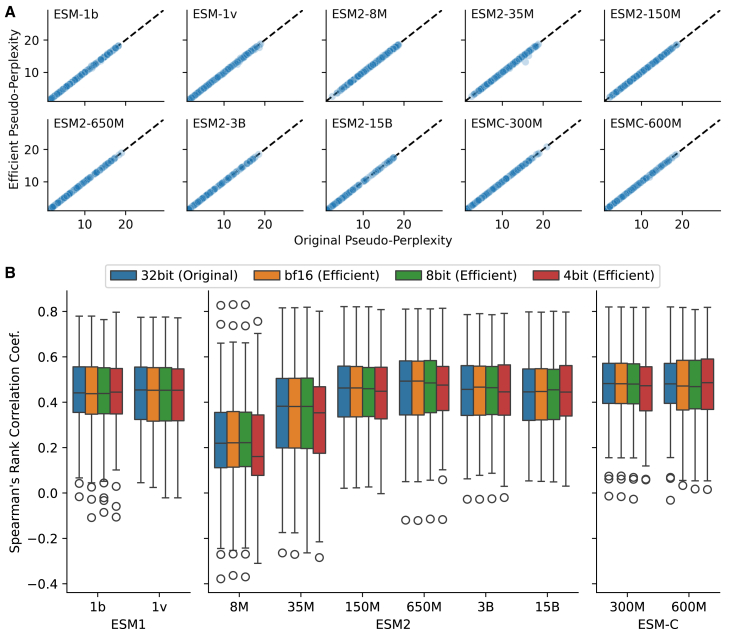
Efficient reimplementation preserves pseudo-perplexity and variant prediction accuracy (A) Pseudo-perplexity comparison between the original models and efficient reimplementations on 500 randomly sampled human proteins. The efficient implementations replicate the original models’ pseudo-perplexity values, confirming the correctness of the reimplementation. (B) Benchmark of protein language models for predicting deep mutational screening (DMS) ground truth. The distribution of Spearman correlation coefficients between the predicted masked margin scores by the protein language models and the DMS measurements from 94 experiments for original implementation with full precision (blue), efficient implementation bfloat16 precision (orange), 8-bit quantization (green), and 4-bit quantization (red). The 300 to 650 million parameter models demonstrate the best performance for variant effect prediction. Quantized 8-bit and 4-bit models perform similarly to unquantized models, except for models with fewer than 150 million parameters, where 4-bit quantization results in reduced performance.

As another validation of correctness, we benchmarked the ESM family—ESM1v, ESM1b, and ESM2—by evaluating how effectively they capture the impact of missense mutations on protein fitness, a key application of PLMs. We used deep mutational scanning (DMS) experimental data from the ProteinGym database as ground truth for 94 human proteins.^47^ The ProteinGym database contains 12 proteins longer than 1024 residues, but the original ESM1b and ESM1v models use learned positional embeddings^57^ with a maximum sequence length of 1024 tokens, requiring sequence truncation for longer proteins. To address this limitation in our implementation, we extended the supported sequence length to 4096 residues in the ESME-1b and ESME-1v models by retraining the positional embedding layer using longer sequences from Uniref. 50 (STAR Methods). Spearman’s rank correlation coefficients between predicted mask margin scores and DMS scores (STAR Methods) demonstrate that our reimplementation produced results comparable to the original implementation on the variant effect prediction task (Figure 2B; Figure S7; Table S4). Weight quantization leads to only a slight performance decrease with 4-bit quantization in smaller models (8M and 35M), while larger models are robust to quantization. Interestingly, model performance improves as the parameter size increases, reaching a peak at 650 million parameters for ESM2, ESM1b, and ESM1v models, with average performance of 46.6 ± 17.5% (SD), 42.7 ± 17.2%, and 43.1 ± 16.7%, respectively. However, performance does not increase further for the larger 3 billion and 15 billion parameter models, staying at 45.2 ± 16.5% and 44.1 ± 16.6%, respectively. The latest ESM-C 300M and 600M models achieve slightly higher correlations of 47.2 ± 16.8% and 46.7 ± 17.3%, respectively. This suggests that the 300 million to 650 million parameter models are optimal for variant effect prediction, and the reduced performance of larger ESM2 models may result from overfitting, potentially leading to an overestimation of the reference amino acids’ probabilities in the masked margin score.

### Benchmarking the model training and fine-tuning

**Figure 3:**
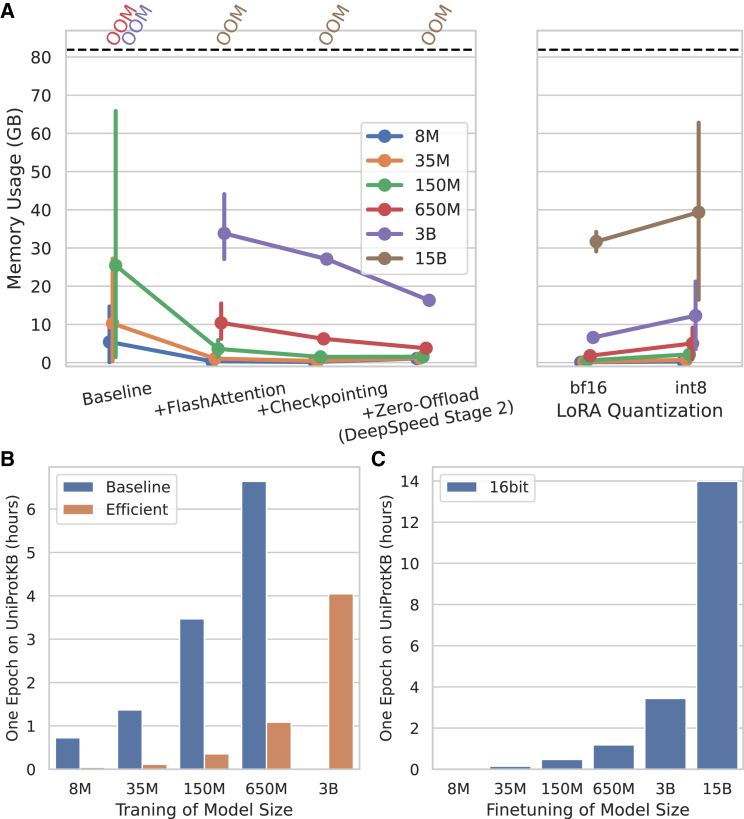
The benchmark of training performance of ESME-2 (A) Memory utilization of model training when the range of training and fine-tuning optimization methods are applied to the ESME-2. OOM indicates an out-of-memory error. (B) One epoch training time of the original ESM2 models and the efficient implementation on the UniProtKB/Swiss-Prot dataset. (C) The runtime of parameter efficient fine-tuning of ESME-2 models for one epoch on UniProtKB/Swiss-Prot.

We also benchmarked the performance of PLMs during training and fine-tuning. We measured memory savings by progressively applying various optimization techniques (Figure 3A; Table S5). Similar to inference, FlashAttention resulted in substantial memory reductions during training. However, training demands more memory than inference due to gradient computation, and Adam optimization states.^58^ Therefore, we integrated training-specific techniques for backpropagation and parameter optimization to further reduce memory consumption. First, activation checkpointing reduces memory usage by recalculating intermediate states during the backward pass instead of storing them, trading runtime for memory savings, thus lowering GPU memory usage from ∼10 GB to 8 GB for the 650 million parameter model and from ∼35 GB to ∼28 GB for the 3 billion parameter ESME models. Another technique, ZeRO-Offload/CPU-Adam, offloads optimization states to CPU memory and performs parameter updates on the CPU (STAR Methods).^59^ This strategy saves ∼39% memory for both the 650 million and 3 billion parameter models. Yet, due to memory limitations, training 15 billion parameters is still not feasible even with all the optimization algorithms with a batch size of 4 proteins.

We measured memory consumption during parameter-efficient fine-tuning by adding LoRA^60^ adapters with a rank of 16 to the query, value, and output layers (Figure 3A). Fine-tuning with these adapters updates only a tiny fraction of the weights, enabling fine-tuning of the 15 billion parameter model (STAR Methods). The largest 15 billion parameter model uses 33 GB of memory, while the remaining models use less than 10 GB. While quantizing frozen model weights during fine-tuning is possible, the current implementations of 8-bit and 4-bit quantization result in increased memory usage as the input token count grows (Figure S8). For example, quantized LoRA fine-tuning with 8-bit quantization increases GPU memory usage from ∼32 GB to ∼40 GB. Thus, unlike inference, quantization during fine-tuning is not memory efficient, highlighting the need for more effective quantization methods for PLMs. Moreover, ZeRO-Offload/CPU-Adam does not significantly reduce memory usage during parameter-efficient fine-tuning, as only the adapters are optimized; thus, offloading optimization states and gradients to CPU memory provide negligible GPU memory savings (Figure S9).

Moreover, we benchmarked the training runtime of ESME-2 after applying all the outlined optimization methods and compared it to the original implementation (Figures 3B; Table S6). Significant speedups were achieved; for instance, one epoch on 571,609 proteins from UniProtKB/Swiss-Prot takes ∼0.04, ∼1.1, and ∼3.4 h for the 8 million, 650 million, and 3 billion parameter models, respectively, using 4 A6000 GPUs. We also estimated the training time for ESM2 models on Uniref. 50 (65,672,468 proteins, totaling ∼18.7 billion tokens) if trained from scratch with our implementation and setup (Figure S10). The original training runs for 500,000 steps with a batch size of 2 million tokens, translating to approximately 53 epochs on Uniref. 50. Based on this estimate, training the 150 million and 650 million parameter models would take 33 and 309 days, respectively.

We further investigated various sequence packing strategies for the ESM-C model using two A100 GPUs with a similar training setup. We found that the original implementation of ESM-C, even with its FlashAttention implementation, is suboptimized due to repeated padding and reshaping at each layer (Figure S11). In contrast, our optimized implementation performs packing only once and uses token-based batching at the dataloader level, improving GPU utilization and reducing training time by up to 53%, thereby demonstrating substantial performance gains with the proposed sequence packing strategy.

Lastly, we measured the runtime of fine-tuning and observed that LoRA fine-tuning is slightly faster compared to the training of all weights (Figure 3C). For instance, fine-tuning 3 billion parameter models takes ∼3.4 h, which is 12.5% faster.

### Finetuning for protein melting point and fitness landscape prediction on the FLIP dataset

**Figure 4:**
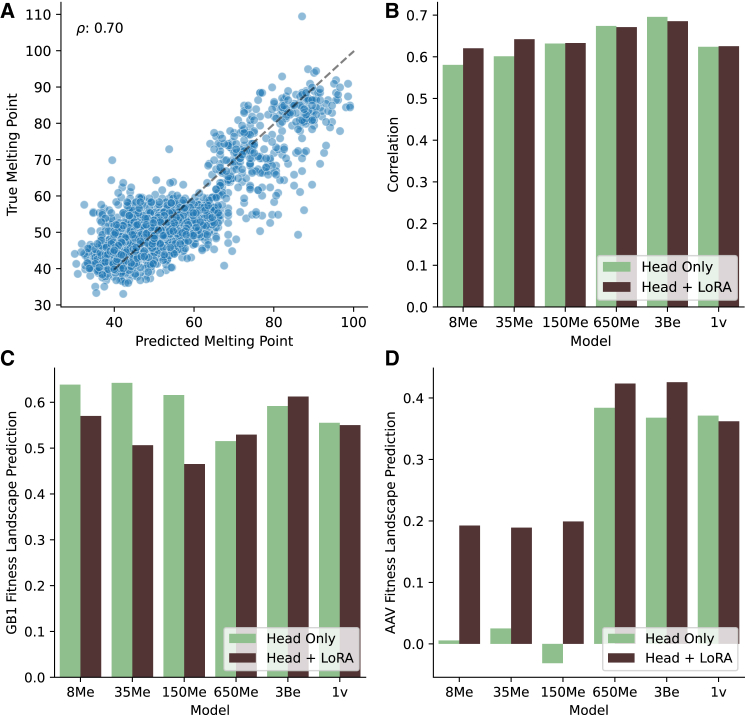
Melting point and fitness landscape prediction with parameter-efficient fine-tuning (A) Correlation between experimentally measured protein melting points from the Meltome Atlas and predictions from the ESME-2 3B model using head-only fine-tuning. The dashed line indicates x = y line. (B) Spearman’s correlation coefficients between observed and predicted melting points, based on head-only (green) and LoRA fine-tuning (brown) of ESME models with different parameter sizes. (C and D) Performance of fitness landscape prediction, measured by Spearman’s correlation coefficients, using head-only fine-tuning (green) and LoRA fine-tuning (brown) for the GB1 and AAV protein datasets.

Annotation of protein function and properties is only available for a limited number of proteins. Thus, leveraging semi-supervised PLMs as foundation models to train task-specific models with limited data are critical. Semi-supervised training of PLMs on diverse sequences enables the capture of latent spaces and protein properties, allowing the model to generalize from limited training samples. To demonstrate this, we fine-tuned ESME-2 using head-only and LoRA fine-tuning to predict the melting point^61^ of proteins and fitness landscape of AAV and GB1 proteins from sequence content using the FLIP benchmarking dataset^28^ (STAR Methods). Based on the hyperparameter tuning on the validation set (Figure S12), adapters with a rank-size of 16 were added to the query, value, and output layers for each transformer block. We achieved a Spearman’s correlation coefficient of ∼70% using head-only fine-tuning of the ESME-2 3B model for melting point prediction, surpassing the state-of-the-art^28^ (Figure 4A; Table S7). LoRA fine-tuning showed improvements for smaller models, boosting performance from 58% to 62% for the 8M model and from 60% to 64% for the 35M model (Figure 4B). The test set consisted of proteins with less than 20% sequence identity to the training set, demonstrating that PLMs can generalize effectively to proteins from unrelated families or remote homologs when predicting protein properties, such as melting point. Furthermore, we achieve a Spearman’s correlation coefficient of 64% on the GB1 protein landscape prediction task using the ESME-2 35M model; however, we observe that LoRA fine-tuning of larger models leads to poorer performance (Table S8; Figure 4C). Interestingly, LoRA fine-tuning leads to a substantial improvement in fitness landscape prediction for the AAV protein, particularly in smaller models. The ESME-2 650M and 3B models with LoRA fine-tuning achieve the best performance, reaching a Spearman’s correlation of 42%, outperforming the FLIP baseline^28^ (Figures 4D; Table S9). Furthermore, we evaluated the efficient implementation of the ESME-1v model across three benchmark tasks. For melting point, GB1, and AAV fitness landscape predictions, we achieved Spearman correlations of 62%, 56%, and 37% using head-only fine-tuning, and 63%, 55%, and 36% with LoRA fine-tuning, respectively. Our findings are comparable to the correlations of 65%, 51%, and 34% reported by the FLIP benchmark for these tasks, respectively**,** which indicates a successful replication of the original results with efficient implementation. These results show that fine-tuning PLMs enables generalization to the prediction of fitness landscapes for unseen protein sequences.

### Homology-based transcription factor prediction

**Figure 5:**
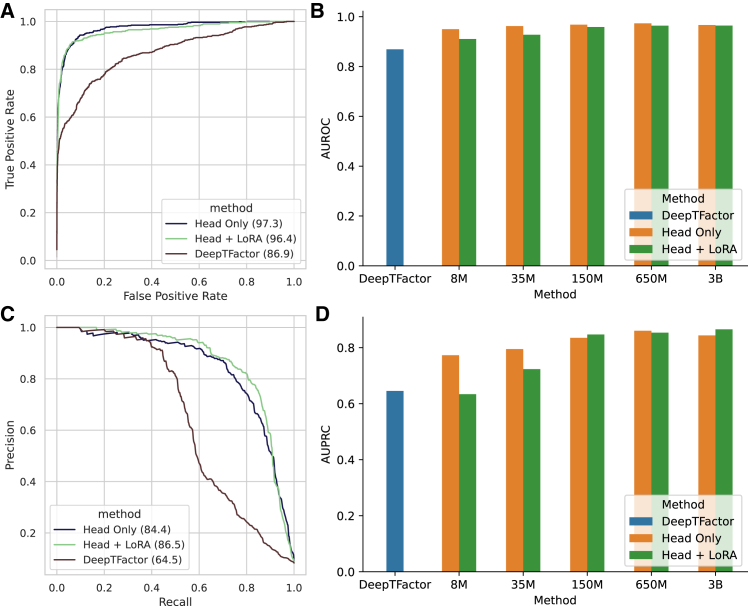
Transcription factor prediction with parameter efficient fine-tuning (A) The receiver operating characteristic curve (ROC) for transcription factor prediction of DeepTFactor, head-only, and LoRA fine-tuned ESME-2 650 million parameter model. (B) The area under the ROC (AUROC) for DeepTFactor, head-only, and LoRA fine-tuned ESME-2 models. (C) The precision-recall curve (PRC) for transcription factor prediction of DeepTFactor, head-only, and LoRA fine-tuned ESME-2 3 billion parameters model. (D) The area under the ROC (AU-PRC) for DeepTFactor, head-only, and LoRA fine-tuned ESME-2 models.

Moreover, we fine-tune the ESME-2 model to predict transcription factors (TFs) using ground truths derived from TF-related GO-terms (STAR Methods). The train and test sets are separated by release time in the database, following the same approach as DeepTFactor. Thus, the prediction task primarily uses homology modeling to estimate protein function. The head-only fine-tuning achieves an area under the receiver operating characteristic curve (AU-ROC) of 97% on the test set, outperforming the state-of-the-art DeepTFactor model,^62^ which achieves 87% AU-ROC (Figures 5A and 5B; Table S10). The LoRA fine-tuning with a rank-size of 16, applied to the query, value, and output layers, achieves slightly higher precision, 87% under the area of the precision-recall curve (AU-PRC) for the 3 billion parameters model, while head-only fine-tuning achieves 84% AU-PRC (Figures 5C and 5D; Table S11). These results demonstrate that PLMs significantly enhance homology-based protein function prediction through a simple fine-tuning procedure.

## Discussion

In this study, significant efficiency improvements are demonstrated in the training, inference, and fine-tuning of PLMs using a combination of advanced techniques such as FlashAttention, activation checkpointing, zero-offload, and low-rank adaptation. Additionally, we introduced dataloaders that effectively utilize sequence packing, enabling transformer models to effectively handle proteins with highly diverse lengths while also significantly extending the batch size. Our efficient reimplementation of the evolutionary sequence model (ESME) achieved up to a 3 to 9-fold reduction in inference time and an 8-fold reduction in memory usage. Moreover, the correctness of our reimplementation is validated by comparing it with the original model through pseudo-perplexity evaluations and missense variant effect prediction, using deep mutational scanning data from ProteinGym as the ground truth for benchmarking. These enhancements enable the use of large-scale PLMs in more resource-constrained environments, such as academic labs.

Our findings underscore the practical utility of these models in various biological contexts. Specifically, the application of PLMs to predict protein properties on the FLIP benchmark dataset and functions—such as melting points and TFs—demonstrates their effectiveness. For instance, fine-tuning ESME-2 for the TF prediction task achieved state-of-the-art performance while training only a small fraction of the model parameters. The LoRA fine-tuning method achieved slightly higher performance, with an AU-PRC of 87%, compared to head-only fine-tuning on TF prediction. In melting point prediction, head-only fine-tuning attained the best performance, with a Spearman’s correlation coefficient of 70%, while LoRA fine-tuning was beneficial only for smaller model sizes. For the GB1 protein fitness landscape prediction, LoRA fine-tuning yields suboptimal results, in contrast to the AAV prediction task, where it achieves the best performance. These results suggest that LoRA fine-tuning can outperform head-only fine-tuning on certain tasks, particularly when the representations of pretrained models are poor, as is often the case with models with smaller parameter sizes. The results indicate that both head-only and LoRA fine-tuning offer distinct advantages, with each achieving the best performance on certain datasets while underperforming on others. LoRA fine-tuning is particularly beneficial when embeddings from the pretrained baseline model are not sufficiently informative; for example, in the case of smaller models on the AAV benchmark. However, LoRA may degrade performance or offer little to no improvement when the pretrained embeddings are already well-aligned with the downstream task, as observed in the GB1 benchmark. Overall, the LoRA adapter weights, trained from a small amount of labeled data, can generate annotations of unannotated protein sequences via homology modeling or *de novo* predictions. Moreover, the adapter weights are highly modular, allowing different adapters to be easily integrated into the base models.

Our observations indicate that quantization techniques effectively save memory by compressing model weights during inference while maintaining comparable results in variant effect prediction. However, their efficacy is limited to models with billions of parameters, and the llm.int8() algorithm doubles the inference runtime, rendering it impractical. However, quantization methods are not as effective during fine-tuning as inference, primarily due to significantly higher memory overhead. We observed that the open-source implementation of quantization algorithms during fine-tuning results in increased memory usage, particularly due to the memory overhead of storing quantization constants, which grow significantly with large input sizes, highlighting the importance of benchmarking before adopting any optimization technique. There are quantization techniques that combine knowledge distillation with quantization.^63^^,^^64^ Although this technique might improve the model performance, further systematic benchmarking of quantization methods is another research avenue beyond the scope of this work.

Overall, the methodologies employed in this study represent a significant advancement toward making PLMs more accessible and practical for a wider range of research applications. The techniques explored are generalizable and not specific to ESM models, and we anticipate that they will be utilized in training the next generation of PLMs. Furthermore, given that the development of optimization techniques for LLMs is still an active area of research, continuous benchmarking and integration of these techniques into PLMs will be essential in the future.

### Limitations of the study

A limitation of the current study is that FlashAttention cannot be used for structure prediction tasks, such as contact map or 3D structure inference, because it does not retain the full attention matrix required for these applications. This restricts the use of the proposed method to sequence-based tasks only. Another limitation is that quantization, while effective at reducing memory usage during inference for large models, significantly increases memory usage during fine-tuning due to the overhead of storing quantization constants and dequantization buffers.

## Resource availability

### Lead contact

Requests for additional information or resources should be addressed to the lead contact, Xiaohui Xie (xhx@uci.edu).

### Materials availability

This study was conducted entirely through computational analyses and did not generate any new materials or reagents.

### Data and code availability

• All data reported in this study are available from the lead contact upon request.
• The original code has been deposited on reproduce GitHub (github.com/uci-cbcl/esm-efficient) and is publicly accessible as of the date of publication. Model weights have been deposited on Hugging Face (huggingface.co/mhcelik/esm-efficient) and are publicly available as of the date of publication.
• Any additional information required to reanalyze the data are available from the lead contact upon request.

## Acknowledgments

This study was funded by the Institute for Precision Health Pilot (IPH) study awards.

## Author contributions

M.H.C. designed the methodology, implemented the software, and performed analyses. X.X. supervised the project. M.H.C. and X.X. wrote the manuscript.

## Declaration of interests

The authors declare no competing interests.

## STAR★Methods

### Key resources table

**Table:** 

REAGENT or RESOURCE	SOURCE	IDENTIFIER
**Software and algorithms**		

FlashAttention	Dao et al.^46^^,^^65^	https://github.com/Dao-AILab/flash-attention
Bitsandbytes	Dettmers et al.^53^^,^^54^	https://github.com/bitsandbytes-foundation/bitsandbytes
ESM1v, ESM1b, ESM2	Lin,^6^ Meier,^16^ Rives^17^	https://github.com/facebookresearch/esm
ESMC	ESM Team^7^	https://github.com/evolutionaryscale/esm
PyTorch	Paszke et al.^66^	https://github.com/pytorch/pytorch
DeepSpeed	Ren et al.^59^	https://github.com/deepspeedai/DeepSpeed
SciPy	Virtanen et al.^67^	https://github.com/scipy/scipy
Scikit-learn	Pedregosa et al.^68^	https://github.com/scikit-learn/scikit-learn

### Method details

In this section, we first outline the transformer architecture of the PLMs. We then described the combination of optimization techniques applied–such as FlashAttention, sequence packing, model quantization, and checkpointing–to enhance the performance of ESM2 during training, fine-tuning, and inference.

#### Protein language models

The attention layers in the model are composed of three key components: embedding layers, transformer blocks, and the language model head. The embedding layers generate embeddings for each amino acid in the input sequence. Positional information of the amino acids is incorporated through learned positional embeddings^69^ in ESM-1b and ESM-1v, while ESM2 and ESM-C employ rotary position embeddings.^70^ The transformer layers, which contain the majority of the parameters of the models, contextualize the input embeddings by capturing interactions between amino acids. These interactions are computed using self-attention, which is formalized as follows:

(Equation 1) $$SelfAttention\left(x\right)=softmax\left(\frac{Q\left(x\right)K\left(x\right)}{\sqrt{d}}\right)V\left(x\right)$$

where query (Q), key (K), and value (V) vectors are used to calculate attention scores between amino ancids, and d is the normalization constant. Each transformer block consists of an attention layer, followed by two feedforward dense layers with GeLU activation,^71^ except for the ESM-C^7^ model, which utilizes SwiGLU^72^ activation and Pre-Layer Normalization^73^ in transformer blocks. Finally, the language model head computes the logits for each amino acid based on the sequence context. The model is trained using a masked language modeling approach, where some amino acids are masked, and the task is to predict the masked amino acids using the surrounding sequence context, optimized with cross-entropy loss.

#### FlashAttention

The memory footprint of standard dot-product attention grows quadratically with sequence length, creating a bottleneck when running models on long protein sequences. FlashAttention, an I/O-aware attention algorithm, addresses this issue by significantly reducing the memory footprint and improving the runtime of attention computations.^46^^,^^65^ Memory access dominates the runtime in the attention calculation. FlashAttention optimizes attention by minimizing the number of read/write operations between the slower GPU high-bandwidth memory (HBM) and the faster on-chip SRAM. Specifically, FlashAttention divides the input key, query, and value matrices into smaller blocks, loading only the necessary blocks into SRAM. All computations are then performed using fused kernels, and intermediate matrices, such as those required for the backward pass, are recalculated as needed to minimize HBM access. By fusing operations together, FlashAttention further accelerates the attention calculation. One limitation of FlashAttention is that it cannot be used for protein structure prediction because structure prediction requires calculating the full attention matrix, which is not stored during the tiled calculation.

#### Sequence packing

We utilized sequence packing to efficiently handle batches of protein sequences with varying lengths. Traditionally, tokens are padded to accommodate flexible input sizes during batch preparation. However, proteins exhibit widely differing sequence lengths, which leads to batches containing a large number of padding tokens and results in wasted computational resources. To address this issue, sequence packing constructs a single long amino acid chain by concatenating all protein sequences in the input batch without any padding tokens and prepares an attention mask that corresponds to the original batch structure. This approach enables batching with a balanced number of tokens, ensuring consistent computational cost in each step and near-full utilization of GPUs. Furthermore, preparing batches with a consistent computational load is essential for distributed data parallelization; thus, sequence packing is critical for scaling computations across multiple GPUs.

#### Retraining the positional embedding layer

We retrained the positional embedding layers of the ESM-1v and ESM-1b models to extend their context size from 1,024 to 4,000 amino acids. To accomplish this, we constructed a training dataset by selecting proteins with lengths between 1,000 and 4,000 amino acids from the Uniref. 50 database,^48^ resulting in 1,755,116 proteins. We reserved 1% of this data as a validation set. During training, we froze all weights except for the positional embedding layer. As a result, only 5,245,440 parameters of positional embeddings were trained. While this approach is suboptimal, since model parameters that may interact with positional embeddings remain frozen, it was chosen to reduce computational cost. We masked 15% of the input tokens and used cross-entropy as the loss function, training the model until convergence.

#### Quantization

As PLMs increase in size, they can consume a significant amount of memory. Rounding model weights to lower-precision numbers can significantly reduce memory usage; for example, half-precision Brain Floating Point (bfloat16) is widely preferred over full-precision float32 due to its lower memory footprint and faster training times.^49^ Model weights can even be represented with lower precision, such as 8-bit and 4-bit formats. For instance, absolute max quantization reduces a matrix to the desired precision by dividing each value by the absolute maximum value, multiplying by the quantization level, and rounding to the nearest integer. However, naive quantization methods introduce errors, thereby significantly degrading accuracy. Therefore, quantization methods specific to language models have been proposed to ensure model performance while reducing memory footprint and runtime. These quantization techniques are helpful during model inference by reducing memory usage and runtime. However, training with quantized weights may not be stable. Nevertheless, quantization can be used during fine-tuning because the quantized model weights are frozen, and only the fine-tuned weights are trained without quantization.

#### 8-bit quantization with LLM.int8

It has been empirically observed that the weights of language models are normally distributed and centered around zero. However, rare outlier features with very large magnitudes can occur. These outlier features are sufficient to shift the quantization scale, thereby introducing large errors in matrix multiplication. To address this issue, LLM.int8^53^ proposes a mixed-precision decomposition methodology that retains outlier features in the original float16 precision and performs calculations in half-precision, while computations for the remaining non-outlier weights are carried out in 8-bit using absolute max quantization. Since the outliers are extremely sparse, storing them in half-precision does not significantly affect the memory footprint while maintaining model performance.

#### 4-bit quantization with QLoRA

QLoRA is an algorithm that efficiently fine-tunes large language models using 4-bit quantization. It employs blockwise quantization, where model weights are divided into blocks that are quantized independently,^54^ effectively containing outliers within each block. This technique stores the quantized weights using the information-theoretically optimal NormalFloat (NF) data type and further reduces memory usage by quantizing the quantization constants through a proposed double quantization method.

#### Activation checkpointing

Model training is significantly more computationally expensive than model inference because the outputs of each layer and the intermediate states needed for backpropagation must be stored during the forward pass to calculate gradients during the backward pass. The accumulation of these intermediate states leads to substantial memory usage in large deep-learning models. Activation checkpointing addresses this issue by significantly reducing GPU memory usage during model training. In this approach, intermediate results are not stored in GPU memory during the forward pass but are recomputed lazily during the backward pass. As a result, activation checkpointing saves memory at the cost of additional computational time.

#### Zero-offload and CPU-Adam

Zero-Offload is a model training strategy that employs CPU offloading to reduce GPU memory usage without significantly degrading model performance.^59^ In the Stage-2 Zero-Offload strategy utilized in this work, the model weights are kept on the GPU, but gradients and optimizer states are offloaded to the CPU after the execution of each layer. Model training requires storing gradients for each parameter and optimization state, such as momentum parameters for the Adam algorithm, thereby multiplying the memory usage per weight. Storing the gradients and optimizer states on the CPU saves GPU memory. However, the offloading process can lead to a slowdown of training because updating the model weights requires transferring gradients and optimizer states between the CPU and GPU. To mitigate the inefficiencies introduced by CPU-GPU communication, Zero-Offload utilizes CPU-Adam,^59^ an algorithm optimized for computation on the CPU. CPU-Adam performs the weight updates on the CPU based on the gradients and optimizer states stored in CPU memory and then transfers the updated model parameters back to the GPU. When this approach is combined with gradient accumulation, where gradients are accumulated over a certain number of steps before updating the model parameters, it minimizes CPU-GPU memory transfer overhead, resulting in minimal efficiency loss during model training.

#### LoRA

Fine-tuning large language models (LLMs) is computationally expensive due to their large parameter sizes. Additionally, information learned during foundational model training might be lost during updates during fine-tuning, a phenomenon known as catastrophic forgetting. Low-Rank Adaptation (LoRA)^60^ addresses these issues by training only a small fraction of the model’s weights, called adapters, during fine-tuning. These low-rank adapters are added to the key, query, and value matrices and are defined as:

(Equation 2) h=Wx+BAx

where $W\in R^{n\times m}$ and $B\setminus \text{in}\;R^{n\times r},A\setminus \text{in}\;R^{r\times m}$ the matrix rank r of the matrixes A and B are much smaller (n≫r,m≫r) than the size of the key, query, and value matrixes. As a result, model weights are frozen, and only the extra adapter weight is trained with LoRA, leading to efficient model training and avoiding catastrophic forgetting. The adapters can be attached and detached from the model, enabling modularity.

#### Pseudo-perplexity

We use pseudo-perplexity as the primary metric to evaluate the goodness of fit of models on protein sequences. Pseudo-perplexity by masking each position in the sequence separately and estimating the probability of the masked token given the unmasked context:

(Equation 3) $$\text{PseudoPPL}\left(x\right)=\exp\left(\frac{1}{T}\sum_{t=1}^{T}-\log\;p\left(x_{t}|x_{\setminus t}\right)\right)$$

where the input sequence of length T and $x_{\setminus t}$ denotes the sequence with the t token masked. The probability $p\left(x_{t}|x_{\setminus t}\right)$ is the predicted probability of the model for the masked token $x_{t}$. Lower pseudo-perplexity indicates lower uncertainty of the model.

#### Variant effect prediction

The PLMs can prioritize potentially pathogenic missense variants by comparing the posterior likelihood of the alternative and the reference amino acids:

(Equation 4) $$v=\log\left(p_{\text{alt}}\right)-\log\left(p_{\text{ref}}\right)$$

where probabilities for the reference ($p_{\text{ref}}$) and alternative ($p_{\text{alt}}$) alleles are predicted by the model. The deep mutational scanning (DMS) experiments provide unbiased experimental ground truth for the benchmark of variant effect prediction on protein fitness. DMS experiments for 92 human proteins were downloaded from the ProteinGym database^47^ and used to validate the correctness of our reimplementation. ESM-1v and ESM-1b models only support sequences up to 1024 amino acids; thus, sequences are truncated to match this length by centering the variants. Similarly, we used this truncation strategy for the original 15 billion parameter ESM2 model to avoid memory error. For ESME models, variant effect predictions were performed with entire protein sequences.

#### Melting point and protein fitness landscapes of GB1 and AAV prediction using the FLIP dataset

Protein language models can be fine-tuned to predict various properties of proteins. To demonstrate this capability, we fine-tuned models to predict the melting points of proteins. We used the Meltome Atlas^61^ as the ground truth for melting points, which contains approximately 48,000 proteins from 13 species. The training and test sets were obtained from the Fitness Landscape Inference for Proteins (FLIP) project,^28^ which splits the data so that sequences in the training and test sets share no more than 20% sequence identity. Additionally, we used the fitness landscapes of GB1 and AAV proteins from the FLIP project as ground truth, where low-fitness sequences were used for training and high-fitness sequences for testing. We employed two fine-tuning approaches: head-only fine-tuning and LoRA fine-tuning for all three datasets.

In head-only fine-tuning, we trained only the regression head while keeping all other layers of the model frozen. For melting point prediction, the model regression consists of two feedforward layers with ReLU activations, taking the average-pooled embeddings as input from the protein language model:

(Equation 5) $$h=\frac{1}{N}\sum_{i=1}^{N}e_{i}$$

(Equation 6) $$y_{\text{pred}}=W_{2}\text{ReLU}\left(W_{1}h+b_{1}\right)+b_{2}$$

where N is the sequence length of the protein, $e_{i}$ is the embedding vector for the i-th token in the sequence, $W_{1}$, $W_{2}$, $b_{1}$, and $b_{2}$ are the weights and biases, and ReLU is the Rectified Linear Unit activation function. In the GB1 and AAV fitness landscape prediction tasks, attention pooling is utilized to aggregate the embeddings E into a vector h before passing it to the regression head:

(Equation 7) $$h=\text{softmax}\left(\frac{C{\left(EW_{k}\right)}^{T}}{\sqrt{d_{k}}}\right)E$$

where h is the final output of the attention pooling layer, C is the learnable matrix of class tokens used to query the attention mechanism, E is the matrix representing the sequence of input token embeddings of a protein, $W_{k}$ is the trainable “key” weight matrix, $d_{k}$ is the dimension of the key vectors which acts as a normalization constant, and softmax is the activation function that transforms attention scores into a weighted average. We implemented an attention pooling layer compatible with sequence packing.

In LoRA fine-tuning, additional adapters were added to the query, key, and value matrices of each transformer layer. For optimization, we used the Adam algorithm with a learning rate of $10^{-4}$ and momentum parameters of 0.9 and 0.999. The learning rate was reduced by 10% every 10 epochs during 100 epochs of training. Mean squared error is used as a loss function. We used a minibatch size of 50,000 tokens with 16 steps of gradient accumulation.

#### Transcription factor prediction

Another downstream application of PLMs is the prediction of protein functions. Specifically, in this work, we fine-tuned PLMs to predict TFs. We annotated proteins in UniProtKB/SwissProt as TFs if they are associated with relevant Gene Ontology (GO) terms (Table S12), using an approach adapted from DeepTFactor,^62^ a state-of-the-art model for TF prediction. We partitioned the data into training and test sets based on the publication date of the proteins in the UniProtKB/SwissProt database: proteins published up to 2021 were used for training, while those published after this date were used for testing. We employed a fine-tuning approach similar to that used for melting point prediction, except that we used the sigmoid function as the final layer of the model head, cross-entropy as the loss function, and upsampled the positive class of TF proteins to balance it with non-transcription factor proteins in each epoch.

### Quantification and statistical analysis

All statistical analyses and model evaluations were conducted in Python (v3.11)^74^ using PyTorch^66^ (v2.3) for model implementation, DeepSpeed^59^ (v0.15) for the ZeRO-Offload strategy, bitsandbytes^55^ (v0.44) for weight quantization, and scikit-learn^68^ (v1.5) and SciPy^67^ (v1.14) for statistical analysis.

Pseudo-perplexity is reported as the primary evaluation metric of model quality for predicting masked amino acids from sequence context. In downstream prediction tasks, we report Spearman’s rank correlation coefficient (ρ) for melting point prediction (Meltome Atlas dataset) and fitness landscape prediction (GB1 and AAV tasks from the FLIP benchmark). For classification tasks (e.g., TF prediction), we report area under the receiver operating characteristic (AU-ROC) and area under the precision–recall curve (AU-PRC). Exact values of test performance, including means, standard deviations (SD), and sample sizes (n), are provided in the main text, figures, and figure legends.

## Supplemental information

Document S1. Figures S1–S12

Table S1. Memory usage for inference with original and efficient ESM2 implementations

Table S2. Inference runtimes of original and efficient ESM2 implementations

Table S3. Perplexity of the original and efficient ESM2 model implementations

Table S4. Correlation between model predictions and experimental deep mutational scanning data for missense variant effects on the ProteinGYM dataset

Table S5. Effect of various optimization strategies on memory usage during model training

Table S6. One epoch training runtime for original and efficient model implementations

Table S7. Melting point prediction performance across different models using head-only fine-tuning

Table S8. Performance of fine-tuned models on GB1 fitness landscape prediction using head-only and Head + LoRA fine-tuning

Table S9. Performance of fine-tuned models on AAV fitness landscape prediction using head-only and Head + LoRA fine-tuning

Table S10. Performance of ESM2 models on transcription factor binding prediction, benchmarked against the DeepTFactor baseline using AUROC

Table S11. Performance of ESM2 models on transcription factor binding prediction, benchmarked against the DeepTFactor baseline using AUPRC

Table S12. Gene Ontology terms used in constructing the ground truth for the transcription factor prediction task
