# Supplementary material for: Efficient inference, training, and fine-tuning of protein language models
Source: iScience. 2025 Sep 3;28(10):113495. doi: 10.1016/j.isci.2025.113495 (PMC12481099; doi:10.1016/j.isci.2025.113495)
Supplement: Document S1. Figures S1–S12 [file mmc1.pdf]

iScience, Volume *28*

Supplemental information

Efficient inference, training, and fine-tuning of protein language models

Muhammed Hasan Çelik and Xiaohui Xie

Supplementary Figures

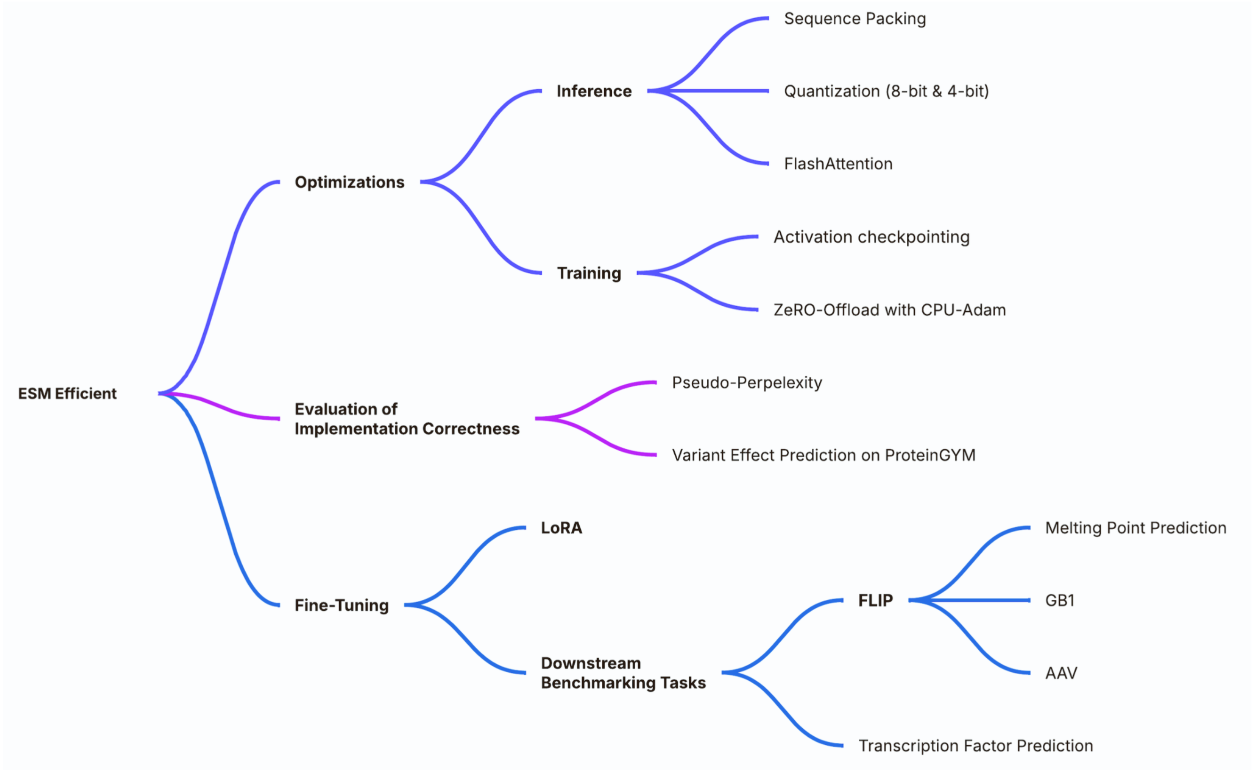

Figure S1: Overview of the methodological framework and analyses presented in this study. We first reimplemented protein language models with efficiency-focused optimizations targeting both inference (e.g., FlashAttention, quantization, and sequence packing) and training (e.g., ZeRO-Offload and activation checkpointing). To verify correctness, we evaluated pseudo-perplexity consistency and variant effect prediction accuracy on ProteinGym. For downstream applications, we explored fine-tuning strategies, including LoRA, and benchmarked performance on tasks such as melting point prediction, fitness landscape modeling (GB1 and AAV from the FLIP dataset), and transcription factor prediction.

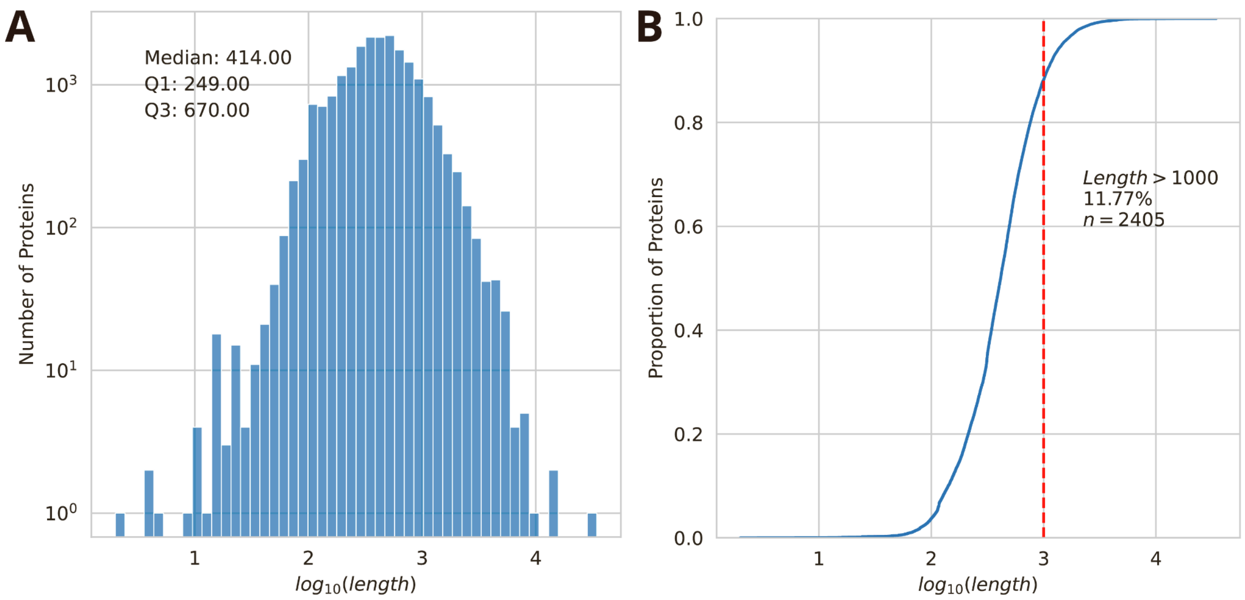

Figure S2: Protein Length Distribution in the Human Proteome.

(A) The length distribution of human proteins in UniProtKB/Swiss-Prot (n=20,435). The median protein length is 414 amino acid residues. Proteins in the lower quartile (Q1) are shorter than 249 residues, while those in the upper quartile (Q3) exceed 670 residues. The longest human protein, Titin (Q8WZ42), consists of 34,350 residues. **(B)** The cumulative density distribution of protein lengths reveals that approximately 12% of human proteins (n=2,045) are longer than 1,000 residues.

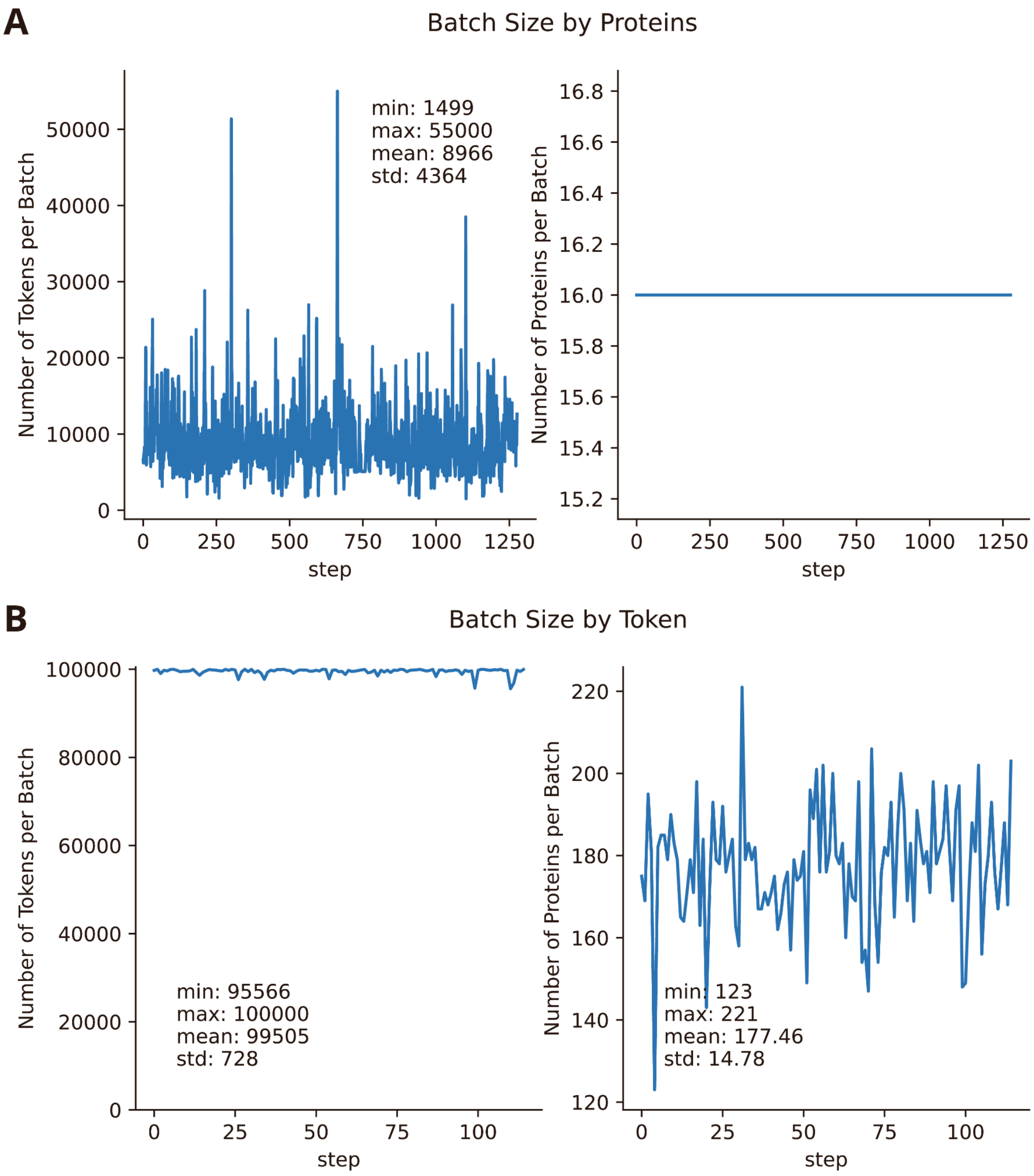

Figure S3: Number of Tokens in a Batch by Batching Technique.

(A) The number of tokens and proteins per batch when batching is based on the number of proteins (batch size of 16 proteins). While the number of proteins per batch is constant, batch sizes vary significantly due to the variable lengths of proteins. Random batching with a fixed protein count from UniProtKB/Swiss-Prot results in an average token size of 8,966 ± 4,364. The largest batch contains 55,000 tokens, while the smallest contains 1,499 tokens. One forward pass on the dataset requires 1,266 steps. **(B)** Batching by the number of tokens (with a maximum of 100,000 tokens per batch) leads to more consistent batch sizes of 99,505 ± 728 tokens. However, the number of proteins per batch fluctuates due to the variation in protein lengths. One forward pass on the dataset requires only 121 steps.

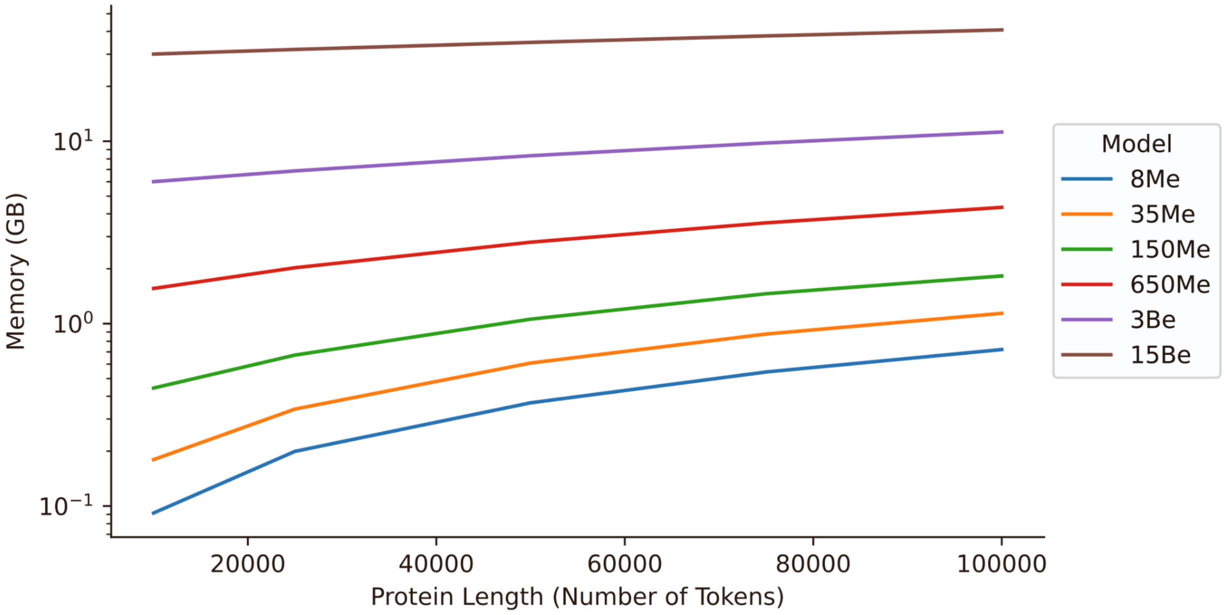

Figure S4: GPU Memory Usage of ESME2 Models.

The sequence context can be extended to 100,000 tokens using the FlashAttention implementation in protein language models, enabling large batch sizes in conjunction with sequence packing.

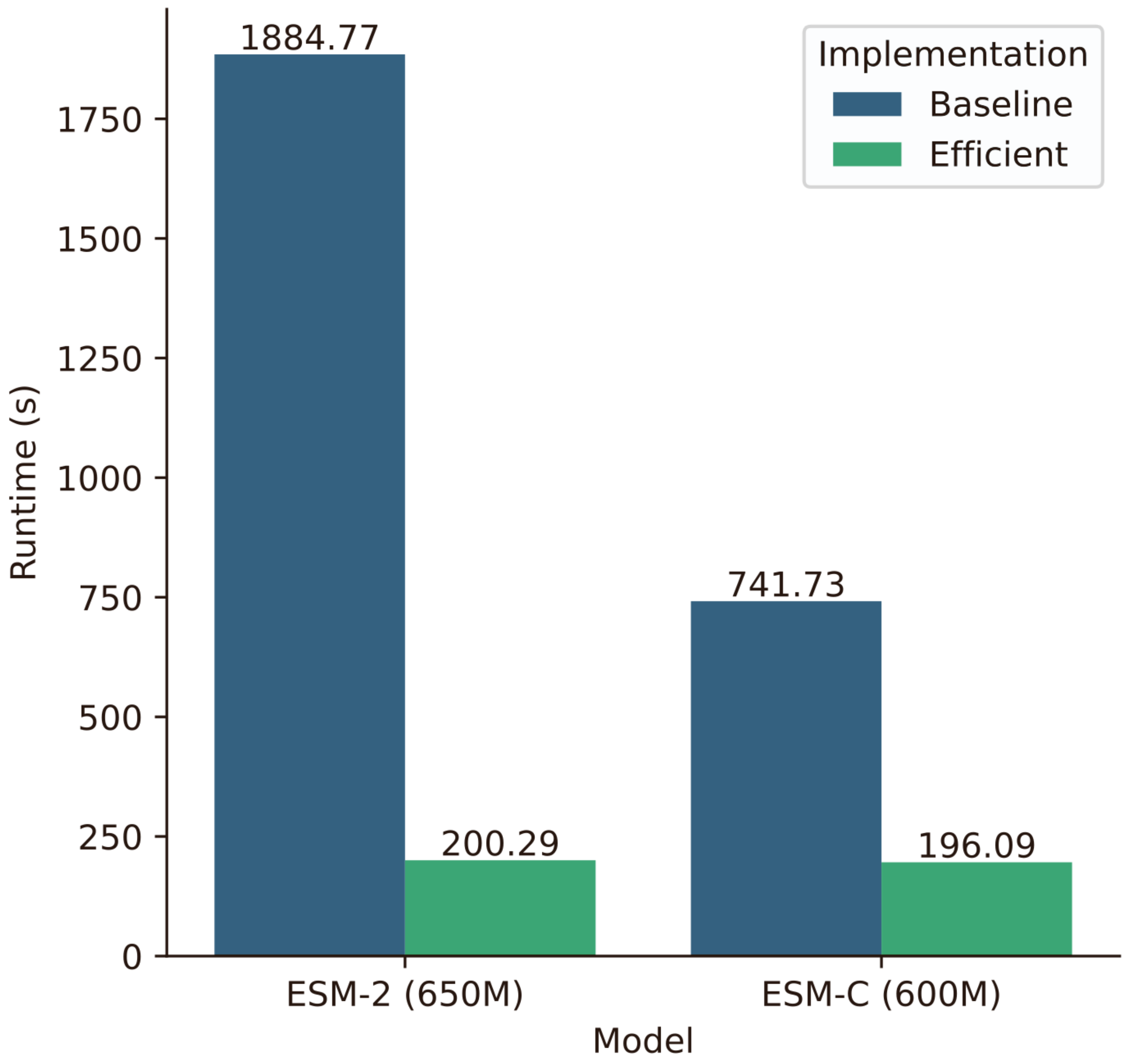

Figure S5: Inference Time on ESM2 and ESM-C Implementations.

A forward pass of the ESM2 model (650M parameters) and the ESM-C model (600M parameters without FlashAttention) on human proteins from the UniProtKB/Swiss-Prot database takes 1,884 seconds and 741 seconds, respectively, using the original implementation. In contrast, employing an optimized implementation with FlashAttention and sequence packing reduces the inference times for the same models to 200 and 196 seconds, respectively.

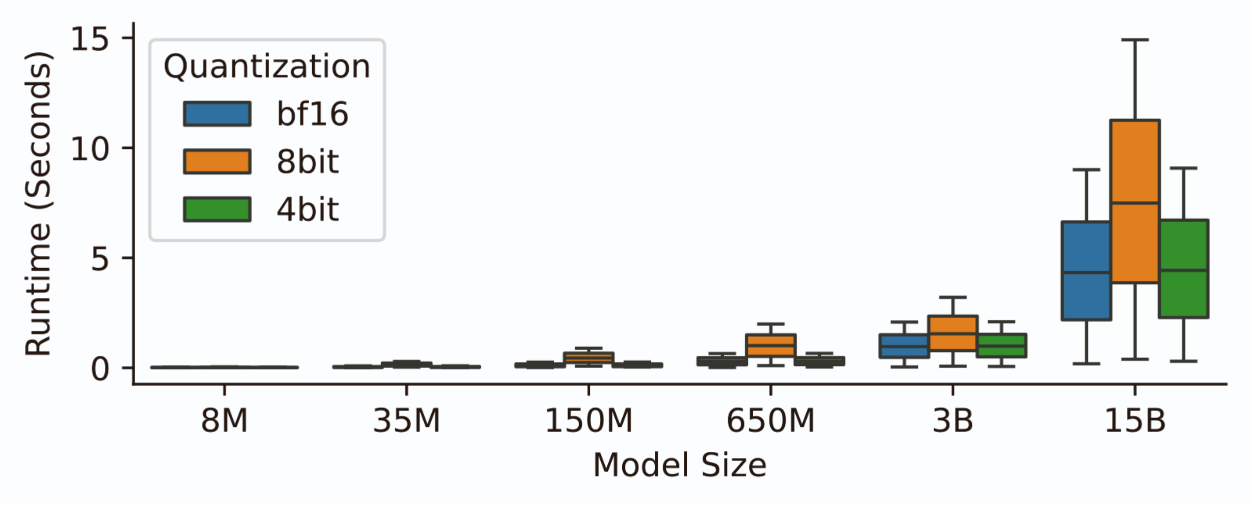

Figure S6: Runtime of Quantized Protein Language Models.

The runtime of models using bfloat16 (brain floating point) and 4-bit quantization is comparable across different model sizes, while 8-bit quantization is slower than bfloat16.

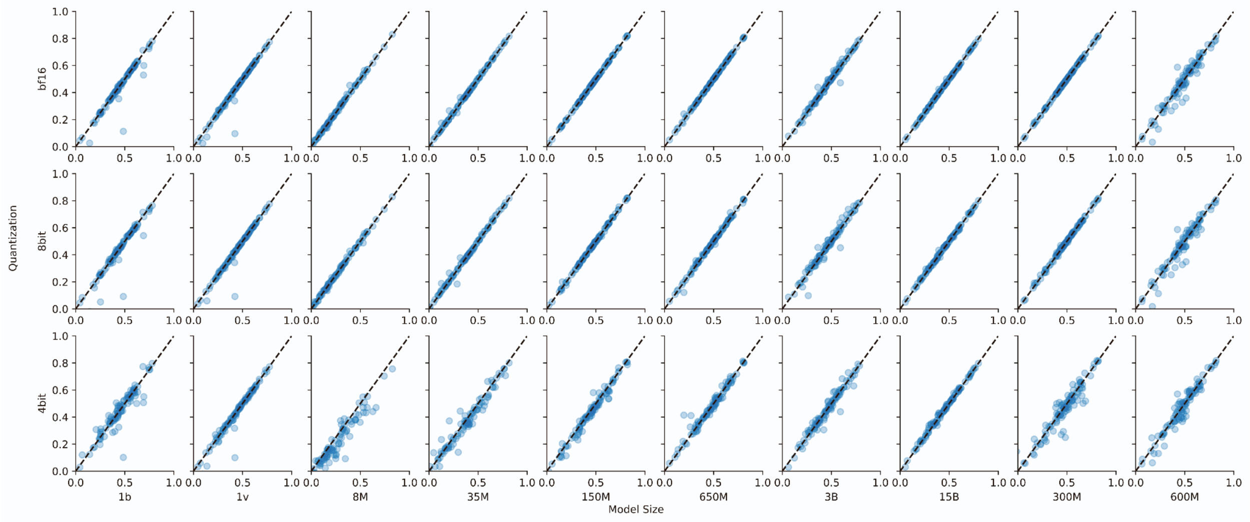

Figure S7: Performance of Quantized Models on Deep Mutational Screening Experiments

Lower-precision and quantized models achieve performance comparable to the full-precision (float32) model across various model sizes for variant effect prediction in deep mutational screening experiments, except for the 8 million and 35 million parameter models, where performance declines with 4-bit quantization.

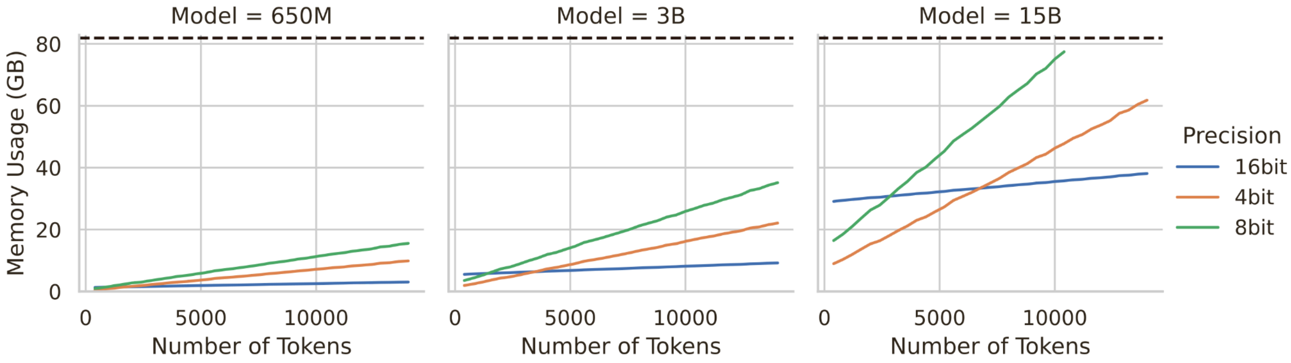

Figure S8: Memory usage of quantized LoRA fine-tuning.

The quantized LoRA fine-tuning uses significantly more memory with increasing token sizes. The quantization of the model weights saves memory, but this saving is offset by the quantization overhead of the models.

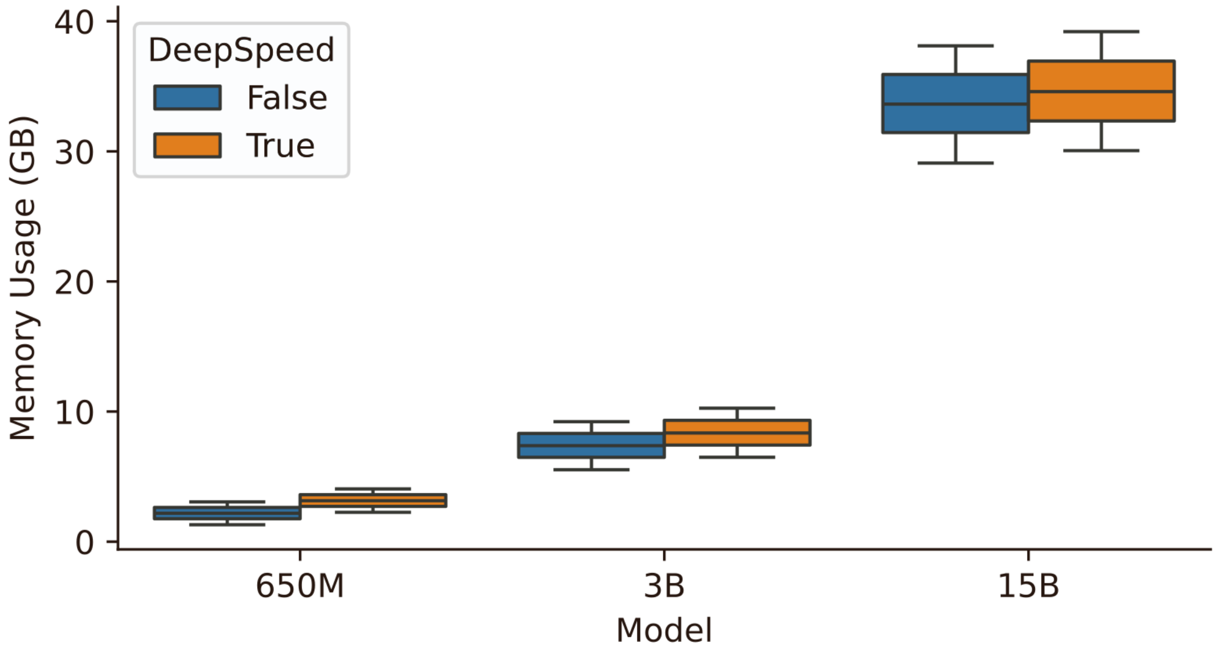

Figure S9: LoRA fine-tuning with/without DeepSpeed

LoRA fine-tuning with DeepSpeed does not reduce memory usage, as only a small fraction of the weights have an optimization state as a result of parameter efficiency.

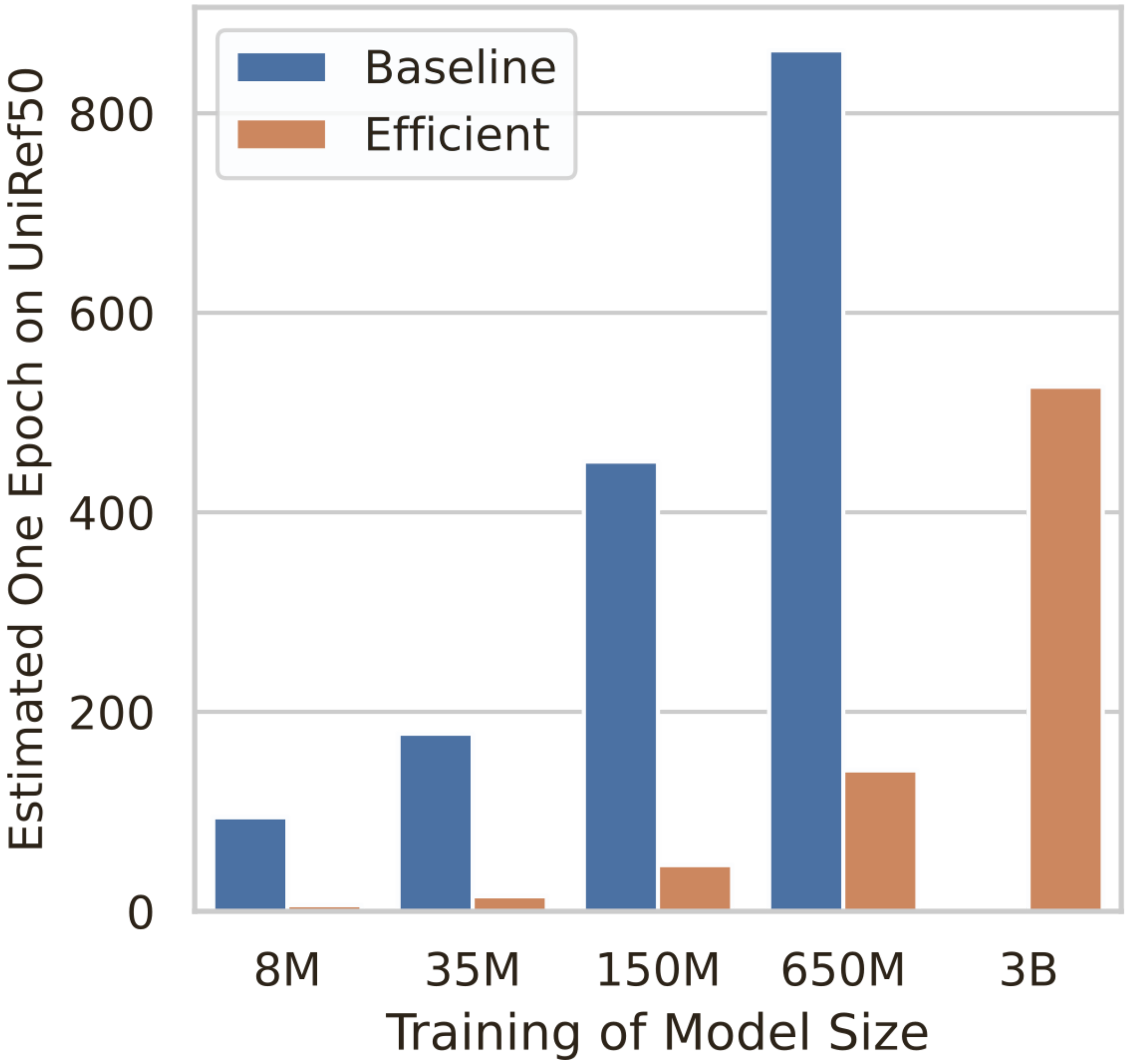

Figure S10: Estimated training time of the ESM2 models.

Efficient implementation of the ESM2 reduces the training time of the ESM2 models 5-11 times across the model sizes. For example, one epoch on UniRef50 training of ESME-2 is estimated as ~180 hours, while the original implementation would take ~900 hours based on the estimated training times on 4 A6000 GPUs.

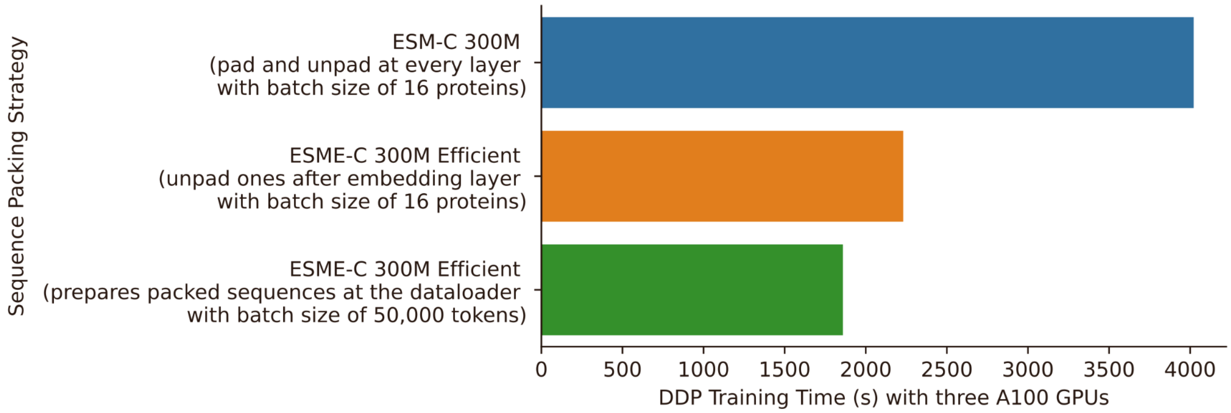

Figure S11: Benchmark of different sequence packing implementations for the ESM-C model. We benchmarked one epoch of training on the UniProtKB dataset using two A100 GPUs with PyTorch Lightning and DDP parallelization. A batch size of 16 was used to evaluate model-level sequence packing, as larger batch sizes lead to memory errors in the original ESM-C implementation. The original ESM-C implementation applies FlashAttention with sequence packing but processes unpacked input sequences, removing padding before each attention layer and reapplying padding afterward. This design introduces memory allocation overhead due to repeated reshaping. Among all methods, the original ESM-C implementation was the slowest due to its inefficient packing strategy (one epoch training time of 4022 seconds). In contrast, the efficient ESM-C implementation unpacks input sequences only in the first layer and performs all remaining computations on packed representations. When batches are prepared by number of proteins, uneven sequence lengths lead to imbalanced workloads across GPUs. In contrast, batching by a fixed number of tokens at the dataloader level improves utilization and reduces this overhead. We further improve the efficiency by introducing dataloader-level token-based packing, which further improves performance by balancing GPU workloads during DDP training. Overall, our optimized sequence packing strategy reduces training time by up to 53%, demonstrating the critical impact of packing methodology on model efficiency and scalability.

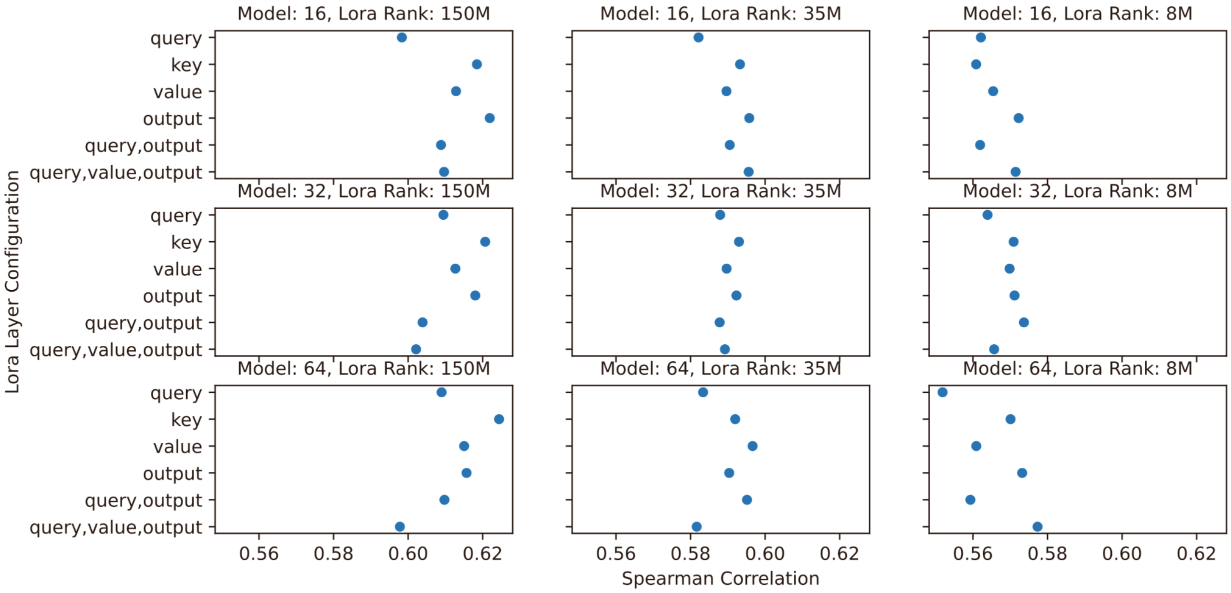

Figure S12: Impact of LoRA hyperparameter choices on validation performance for protein melting point prediction. We systematically evaluated LoRA rank values (16, 32, and 64) and adapter placements (query, key, value, and output projections). The results show that rank size has a minimal effect on validation accuracy, with performance varying by approximately 2% across configurations. These findings suggest that low-rank adapters (e.g., rank 16) are sufficient for parameter-efficient fine-tuning with only minor trade-offs in performance.
